# Chemical Profiles and *In Vitro* Cholinesterase Inhibitory Activities of the Flower Extracts of *Cassia spectabilis*

**DOI:** 10.1155/2023/6066601

**Published:** 2023-02-28

**Authors:** Suciati, Erlinda R. Laili, Wiwied Ekasari, Kuatman, Nitra Nuengchamnong, Nungruthai Suphrom

**Affiliations:** ^1^Department of Pharmaceutical Sciences, Faculty of Pharmacy, Universitas Airlangga, Surabaya 60115, East Java, Indonesia; ^2^Center for Natural Product Medicine Research and Development, Institute of Tropical Diseases, Universitas Airlangga, Surabaya 60115, East Java, Indonesia; ^3^Purwodadi Botanic Garden, Department of Scientific Collection Management, National Research and Innovation Agency (BRIN), Pasuruan 67163, East Java, Indonesia; ^4^Science Laboratory Center, Faculty of Science, Naresuan University, Phitsanulok 65000, Thailand; ^5^Department of Chemistry, Faculty of Science and Center of Excellence for Innovation in Chemistry, Naresuan University, Phitsanulok 65000, Thailand

## Abstract

**Background:**

*Cassia spectabilis* is a flowering plant containing various metabolites that provide potential for pharmacological activities. The current study aimed to investigate the ethanolic and water extracts of *C. spectabilis* as cholinesterase inhibitor as one of the target treatments for Alzheimer's disease. The chemical composition of the extracts was also studied to determine which components are responsible for the bioactivity.

**Methods:**

The cholinesterase inhibitory activity assay was carried out by the modified Ellman's method against acetylcholinesterase (AChE) and butyrylcholinesterase (BChE). LC-MS/MS analysis was carried out to investigate the chemical profiles of the extracts, followed by a molecular networking study by GNPS.

**Results:**

Both extracts showed inhibition against AChE and BChE in a dose-dependent manner, with the higher potency exhibited by the ethanolic extract with IC50 values of 7.88 and 3.78 *μ*g/mL. The chemical analysis and molecular networking study of the flower extracts revealed similarity between the ethanolic and water extracts. Piperidine alkaloids were identified in both extracts, while the sphingolipid compounds were found in the ethanolic extract.

**Conclusion:**

The water and ethanolic extracts of *C. spectabilis* flowers displayed potency for Alzheimer's disease treatment. The presence of piperidine alkaloids in the extract may be responsible for the cholinesterase inhibitory activity. The higher potency of the ethanolic extract compared to the water extract is possibly due to the higher amount of piperidine alkaloids in the ethanolic extract. Further study is needed to quantify the concentration of alkaloids in the extracts.

## 1. Introduction

Alzheimer's disease (AD) is a progressive neurologic disorder that mainly affects the elderly population. The early sign of AD is mainly characterized by mild memory declines, such as forgetting recent events or conversations. As the disease progress, it will develop into severe memory loss and language impairment, as well as behavioral and social changes that will eventually affect the person's ability to carry out daily tasks [1, 2]. It is estimated that more than 50 million people are suffering from AD, and the number is expected to increase to 115.4 million in 2050 [3, 4].

Multiple factors are involved in the pathogenesis of AD, including the deficiency of the neurotransmitter acetylcholine (ACh). Two cholinesterase enzymes are involved in regulating ACh in the brain, namely acetylcholinesterase (AChE) and butyrylcholinesterase (BChE). Both enzymes hydrolyze ACh into choline and acetate [5]. In the healthy brain, the regulation of ACh is mainly controlled by AChE compared to BChE. However, in a patient with AD, the BChE level increases from 0.6 in the normal brain to as high as 11 in the cortical areas affected by the disease [6, 7]. As a consequence, the regulation of ACh is dependent on the activity of the BChE enzyme. Therefore, cholinesterase enzyme inhibition becomes one of the target therapies for AD. The use of cholinesterase inhibitors has shown positive effects in improving the cognitive function of AD patients [8, 9].

*Cassia spectabilis*, synonym *Senna spectabilis* (family *Fabaceae*) is a flowering plant distributed in tropical and sub-tropical regions [10]. The flowers with unsymmetrical golden-yellow corolla located terminal. Various parts of this plant have been used as folk medicine in Indonesia, Brazil, and Thailand. The leaves are used as laxatives and purgatives. Other traditional uses are for treating skin diseases, edema, as well as poisoning, and protozoic infection of the gut [11]. The infusion of the leaves is traditionally used for insomnia and anxiety [12]. Chemical investigation revealed the presence of alkaloids, terpenoids, flavonoids, anthraquinones, and steroids from the leaves, flowers, fruits, seeds, stems, and roots of *C. spectabilis* [11, 13, 14]. The plant has been reported as anticonvulsant, antimicrobial, antinociceptive, and anti-inflammatory agents [15–20]. In the previous study, we investigated the potency of the ethanolic and water extracts of *C. spectabilis* leaves and stems as antioxidant and cholinesterase inhibitors. The leaves and stems extract of *C. spectabilis* exhibited significant cholinesterase inhibitory activities against AChE and BChE as well as moderate antioxidant activities [21, 22]. In the development of herbal medicinal product for the treatment of Alzheimer's disease from *C. spectabilis*, information on the extraction solvent that gives the optimum potency as a cholinesterase inhibitor is needed. Traditional use of the plant as an infusion and decoction has been documented [12]. Meanwhile, studies on the methanolic extract of *C. spectabilis* flower as a cholinesterase inhibitor has been reported [23]. Therefore, it is interesting to know which extraction solvents that will give the best cholinesterase inhibitory activity. The current study evaluated the cholinesterase inhibitory activities and the phytochemical profile of the water and ethanolic extracts of *C. spectabilis* flowers. We hypothesize that different extraction solvents will have an effect on the chemical profiles and the cholinesterase inhibitory activities. To the best of our knowledge, there is no report on the effect of different extraction solvents on the cholinesterase inhibitory activity and the chemical profile of *C. spectabilis* flowers.

## 2. Materials and Methods

### 2.1. Plant Materials

The flowers of *Cassia spectabilis* were collected from Purwodadi Botanic Garden and Mliwis Mountain, Pasuruan, East Java, Indonesia, in February and March 2022. A dried plant specimen was kept at the Faculty of Pharmacy, Universitas Airlangga. The plant was identified by Eka Karya Botanical Garden Characterization Laboratories, National Research, and Innovation Agency with identification letter number: ELSA 34121.

### 2.2. Chemicals

Acetylcholinesterase from electric eel type VI-S, butyrylcholinesterase from equine serum, acetylthiocholine iodide (ATCI), butyrylthiocholine iodide (BTCI), 5,5′-dithiobis[2-nitrobenzoic acid] (DTNB), bovine serum albumin (BSA), tris buffer, and galantamine were purchased from Sigma–Aldrich. The organic solvents (methanol and ethanol) and reagents used in the analysis were of analytical grades.

### 2.3. Extraction

The flowers of *C. spectabilis* were dried in the shades for approximately seven days and then grounded using an electrical blender. The ethanolic extract of the flower was prepared by adding ethanol (500 mL) to the dried flower powdered (88.2 g), and the mixture was allowed to stand for 24 h, followed by vacuum filtration. Then the residue was re-extracted with ethanol using the same procedure twice. The collected filtrate was evaporated under vacuum to obtain a dark brown extract (7.0 g). For water extract preparation, the dried flowers (80.3 g) were soaked in water (500 mL) in an ultrasonic chamber for 30 minutes, followed by filtration. Then the residue was re-extracted with water using the same procedure twice. The filtrates were evaporated in a rotary evaporator to yield a dark brown water extract (15.0 g).

### 2.4. Cholinesterase Assay

The assay was carried out according to the modified Ellman's method, as described in the previous publications [22, 24]. The extracts were dissolved in methanol to make 10 mg/mL and diluted with water to obtain 1 mg/mL. These samples were serially diluted to get a range of sample concentrations of 0.01–300 *μ*g/mL. Sample solutions were added to a 96-well microplate (25 *μ*L), followed by the addition of 1.5 mM ATCI (25 *μ*L) as a substrate for the AChE enzyme, and BTCI (25 *μ*L) as a substrate for the BChE enzyme. The substrate was then hydrolyzed using 3 mM DTNB (125 *μ*L), tris-buffer (50 *μ*L), and AChE or BChE enzymes (25 *μ*L) 0.22 *μ*/mL. Before measurement, the solutions were shaken for 30 s in a microplate reader (Thermo Scientific Multiskan FC). The yellow color from the product, 5-thio-2-nitrobenzoate, was measured at 405 nm every 5 s for 2 min. Every experiment was carried out in triplicates. Methanol 10% was used as a control. The enzyme activity was calculated as a percentage of the velocity of the sample compared with the negative control. The inhibitory activity was calculated based on the equation.

(1) $$\begin{array}{c} \%Inhibition=\frac{\left(Vcontrol-Vsample\right)}{Vcontrol}\times 100\text{,} \end{array}$$

where *V*: mean velocity.

### 2.5. LC-MS/MS and Global Natural Product Social (GNPS) Molecular Networking

The extracts were dissolved in methanol (10 mg/mL), sonicated, and then filtered with a 0.22 *μ*m membrane. Chromatographic separation was performed using an Agilent 1260 Infinity Series HPLC system with an auto-sampler fitted with an analytical C-18 column (Phenomenex Luna C18(2), 150 × 4.6 mm, 5 *μ*m, USA). A 10 *μ*L sample was injected into the LC system and eluted with a combination of water containing 0.1% formic acid (A) and 5% v/v acetonitrile with 0.1% formic acid (B). A linear gradient from 5 to 95% B in 30 mins, and hold on at this ratio for 10 mins. The postrun was set for 5 mins before starting a new injection. The solvent flow rate was set to 0.5 mL/min. The mass analysis was performed using a QTOF 6540 UHD accurate mass spectrometer. The analysis parameter was carried out in a positive mode with spectra acquired over a mass of m/z 100–1,000 amu. The ESI-MS condition parameters were as follows: capillary voltage +3,500 V; drying gas (N_2_) 7 L/min; dry gas temperature at 350°C; and nebulizer pressure at 30 psig. Fragmentations were performed using auto MS/MS experiments with collision energies at 10 V, 20 V, and 40 V. Compounds identification was carried out based on the MS data, MS/MS fragmentation profiles, and molecular formula proposed by the Agilent MassHunter that were compared with the literature data and some databases, such as the Human Metabolome and MetFrag, with a maximum error of 5 ppm was accepted. A GNPS analysis was carried out on the LC-MS/MS data. The LC-QTOF-MS/MS data from Agilent MassHunter data files (.d) were converted to mzXML file format using MSConvert software. The data were then transferred to the GNPS server (gnps.ucsd.edu) to generate the chemical networking map (ID = 011c1f464d314d74bdf209c18a39f4a5) [25]. The GNPS analysis workflow using the spectral clustering algorithm with a cosine score of 0.7 and a minimum of 4 matched peaks in the fragmentation spectrum. The molecular networking data were visualized with Cytoscape software version 3.9.1. A ball-and-stick layout where nodes represent parent mass and cosine score was reflected by edge thickness [25, 26].

## 3. Results and Discussion

### 3.1. Cholinesterase Inhibitory Assays

**Figure 1:**
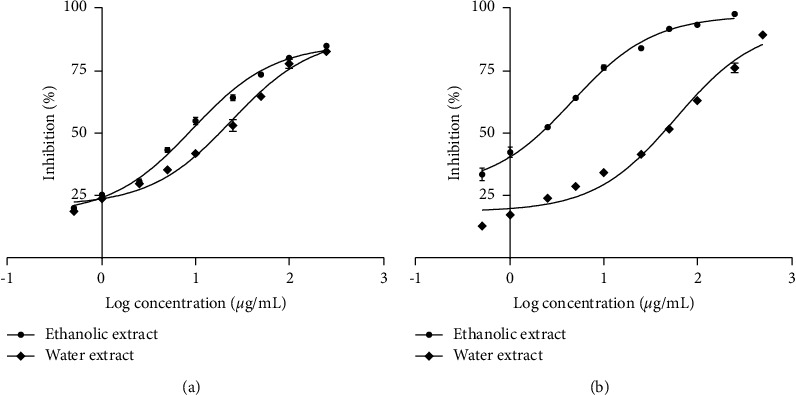
Concentration-dependent response of *C. spectabilis* flower extracts against AChE (a) and BChE (b); each value is expressed as means ± SEM (*n* = 3).

The ethanolic and water extracts of *C. spectabilis* flowers were screened against the AChE and BChE using a colorimetric method developed by Ellman et al. [27]. In this assay, AChE and BChE enzymes will hydrolyze the substrates acetylthiocholine iodide (ATCI) and butyrylthiocholine iodide (BTCI), respectively. As a result, a product thiocholine will form, which then reacts with the Ellman's reagent (DTNB) to form a yellow-colored product 5-thio-2-nitrobenzoate that can be monitored using a spectrophotometer at 405 nm. The presence of the AChE or BChE inhibitors will prevent the hydrolysis of ATCI and BTCI so that the yellow-colored product will not be formed [27]. Various concentrations of *C. spectabilis* extracts were prepared to evaluate the dose-response mode and the fifty percent inhibitory activity (IC50) of the extract. The results (Table 1) shows that the ethanolic extract of *C. spectabilis* flower demonstrated high inhibition against both AChE and BChE with IC50 values of 7.88 *μ*g/mL and 3.78 *μ*g/mL, respectively. Meanwhile, the water extract inhibited moderate inhibition against both enzymes.  Figure 1 indicates that both extracts inhibited AChE and BChE enzymes in a dose-dependent manner.

Inhibition of AChE and BChE enzymes prevents the hydrolysis of acetylcholine and increases the level of ACh in the brain. The association between BChE enzyme and accumulation of neuritic plaques and fibrillar A*β* plaques as one of the pathologic hallmarks of AD has been reported. Therefore, inhibition against BChE serves a dual role, increasing acetylcholine levels and inhibiting fibrillar A*β* deposition [7].

Several plant species from the genus *Cassia* have been reported as cholinesterase inhibitors. Azman et al. have screened the potency of *C. timorensis*, *C. grandis*, *C. fistula*, *C. alata*, and *C. spectabilis*. Amongst the plants tested, extracts from the leaves, stems, and flowers of *C. timorensis* showed significant inhibition against AChE [28]. We have investigated the cholinesterase inhibitory activities of various extracts from the leaves and stems of *C. spectabilis* that showed the potency of the leaves and stems extracts against both AChE and BChE enzymes [22]. Franca et al. reported the AChE inhibitory potency of the methanolic extract of *C. spectabilis* flower using a TLC bioautography method, which however, cannot determine the activity quantitatively [23]. Studies on the effect of *C. spectabilis* on central nervous system disorders have been documented. The ethanolic extract of *C. spectabilis* leaves showed anticonvulsant and sedative activity in mice [12]. Further study by Nkamguie Nkantchoua et al. investigated the anticonvulsant activity of *C. spectabilis* leaves decoction. The oral administration of the decoction can completely inhibit seizure in mice induced by maximal electroshock, antagonizes completely seizures induced by pentylenetetrazole, and partially inhibit seizure induced by bicuculline [29]. Silva et al. reported that iso-6-cassine and iso-6-spectaline isolated from *C. spectabilis* possess anticonvulsant and depressant effects on mice [19, 30]. Oral administration of iso-6-cassine and iso-6-spectaline at a dose of 1.5 mg/kg BW and 1.0 mg/kg BW, respectively, can significantly decrease motor activity of the animal on rota-rod apparatus. Both compounds were capable of promoting an increase in latency for the development of convulsion triggered by picrotoxin.

### 3.2. Chemical Profiles and Molecular Networking Study

**Figure 2:**
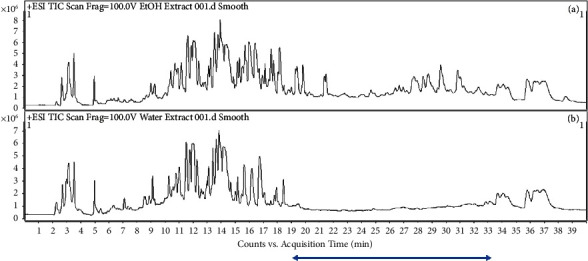
Total ion chromatogram of the ethanolic (a) and water (b) extracts of *C. spectabilis* flower. The blue arrow refers to the region in which the extracts show different TIC profile.

**Figure 3:**
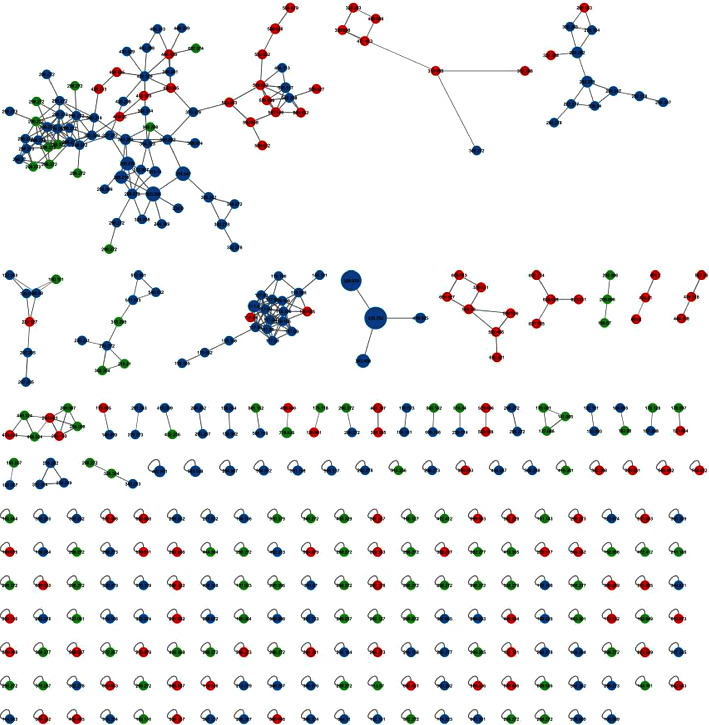
Molecular networking of the compounds from the ethanolic and water extracts of *C. spectabilis* flower.

The chemistry of ethanolic and water extracts of *C. spectabilis* was explored using an LC-MS/MS instrument. The total ion chromatograms (TIC) of the extracts were compared (Figure 2), which are very similar except at RT 19–33 minutes. The GNPS molecular networking (Figure 3) shows the cluster of compounds present in the ethanolic and water extracts. The red dots represent compounds identified in the ethanolic extract, the green dots for compounds present in the water extract, and the blue dots for compounds identified in both the ethanolic and water extracts. As can be seen in Figure 3, the majority of compounds can be detected in both extracts; this data is in agreement with the TIC profile of the extracts.

**Figure 4:**
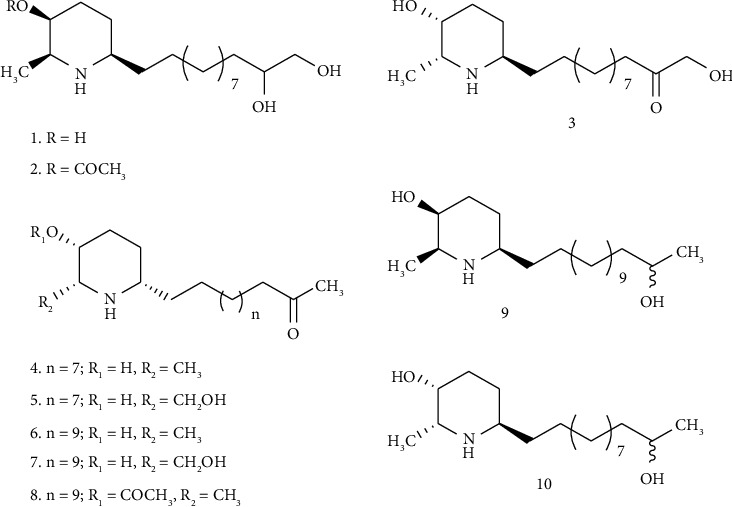
Piperidine alkaloids identified in the ethanolic and water extracts of *C. spectabilis* flower.

Alkaloid has been reported as the major class of compound that shows promising activity as a cholinesterase inhibitor [2, 31]. Therefore, in the present study, we focus on identifying alkaloids in the extracts. The identified alkaloids in the ethanolic and water extracts of *C. spectabilis* flower are presented in Tables 2 and 3. There are 22 compounds identified in the ethanolic extract and 20 in the water extract. Fifteen compounds were detected in both water and ethanolic extracts, including the well-known piperidine alkaloids that have been reported from *Cassia spp*., such as leptophyllin A (**1**), 3-*O*-acetylleptophyllin A (**2**), and leptophyllin B (**3**) that first isolated from *Cassia leptophylla* [32] and later from *Cassia spectabilis* [13]. Other piperidine alkaloids detected in both extracts were (−) cassine (**4**), (−)-7-hydroxycassine (**5**), (−)-spectaline (**6**), (−)-7-hydroxyspectaline (**7**), (−)-3-*O*-Acetylspectaline (**8**), (−)-spectalinine (**9**), and (−)-6-iso-carnavaline (**10**) (Figure 4). Based on the TIC profiles, the difference between the water and ethanolic extracts is the peaks at 25–31 mins region, from which sphingolipids were detected.

Selegato et al. have summarized secondary metabolites reported from various parts of *C. spectabilis* [13]. The flower contains alkaloids, pentacyclic triterpenes, steroids, pyrones, anthraquinones, and phenylpropenoic acid. Piperidine alkaloids have been reported as the major constituent in the aerial part of *C. spectabilis*. Viegas et al. reported the isolation (−)-3-*O*-acetylspectaline, (−)-7-hydroxyspectaline, iso-6-spectaline, and (−)-spectaline from the flower of *C. spectabilis* [33]. (−)-Spectaline (**6**), (−)-7-hydroxyspectaline (**7**), (−)-3-*O*-acetylspectaline (**8**) were detected in both the water and ethanolic extracts in this study. However, iso-6-spectaline was not detected. Cholinesterase inhibitory activities of natural and semisynthetic piperidine alkaloids have been reported. (−)-3-*O*-acetylspectaline hydrochloride and (−)-3-*O*-acetylcassine hydrochloride prepared from (−)-cassine and (−)-spectaline have shown inhibition against rat brain AChE and BChE as well as in the *in vivo* spatial memory assay [34, 35]. Further analysis using molecular docking of these compounds showed that (−)-3-*O*-acetylcassine hydrochloride significantly interacts with the catalytic triad of AChE [36]. Castro et al. also reported two semisynthetic compounds, LASSBio-767 [(−)-3-*O*-acetylspectaline] and LASSBio-822 [(−)-3-*O*-tert-Boc-spectaline] derived from (−)-spectaline as CNS-selective noncompetitive cholinesterase inhibitors [37]. Silva et al. synthesized 12 analogous from (−)-cassine and (−)-spectaline, and four of the semisynthetic compounds significantly inhibited BChE in the *in vitro* assay [38]. Our previous study showed that the ethanolic extract of *C. spectabilis* leaves contains piperidine alkaloids and displays significant cholinesterase inhibitory activities [21, 22]. Based on the various reports on the potency of piperidine alkaloids as cholinesterase inhibitors [34–38], it can be suggested that the cholinesterase inhibitory activities of the ethanolic and water extracts of *C. spectabilis* flowers in this study possibly due to the presence of the piperidine alkaloids detected in the samples. The higher cholinesterase inhibitory activity of the ethanolic extract compared to the water extract may occur due to the higher amount of piperidine alkaloids in the ethanolic extract. To quantify the concentration of alkaloids in the extracts, additional research is required.

## 4. Conclusions

The ethanolic extract of *C. spectabilis* flowers demonstrated higher potency as cholinesterase inhibitors compared to the water extract. Despite the qualitative analysis of the component in the extract, the presence of piperidine alkaloids in the extract may be responsible for the bioactivity. Based on this study, the ethanolic extract of the *C. spectabilis* flower can be a good candidate for the development of herbal medicine for Alzheimer's disease. Future works could focus on the quantitative analysis of the alkaloid in the extract and the *in vivo* study using the Alzheimer's disease animal model in the behavioral assays.

## Figures and Tables

**Table 1:** Cholinesterase inhibitory activities of *C. spectabilis* flower extracts and standard.

Samples	IC50 (*μ*g/mL)^a^	
	AChE	BChE
Ethanolic extract	7.88 ± 0.14	3.78 ± 0.10
Water extract	27.29 ± 1.14	47.10 ± 0.59
Galantamine	0.20 ± 0.01	1.33 ± 0.02

^a^Data presented as mean ± SEM of three experiments, each done in triplicates.

**Table 2:** LC-MS/MS analysis of the ethanolic extract of *C. spectabilis* flower.

RT^a^ (mins)	(M + H)^+^	Product ions (m/z)	Formula	Exact mass	Diff (ppm)	Proposed compounds
3.162	138.0548	94.0645, 92.0488, 78.0332, 65.0380, 53.0380	C_7_H_7_NO_2_	138.0550	1.12	6-methylpicolinic acid
3.481	130.0858	103.8087, 84.0803, 67.0534, 56.0491	C_6_H_11_NO_2_	130.0863	3.50	Pipecolic acid^*∗*^
7.422	230.1739	212.1630, 194.1522, 95.0849, 70.0645	C_12_H_23_NO_3_	230.1751	5.08	7-(5,6-dihydroxypiperidin-2-yl)heptan-2-one^*∗*^
8.63	244.1905	226.1789, 208.1679, 185.0062, 95.0846, 70.0647	C_13_H_25_NO_3_	244.1097	0.90	7-(5-hydroxy-6-(hydroxymethyl)piperidin-2-yl)heptan-2-one^*∗*^
9.232	332.2790	314.2672, 296.2566, 260.2368, 236.2351, 70.0647	C_18_H_37_NO_4_	332.2795	1.61	Batzellaside A^*∗*^
9.246	346.2572	328.2466, 310.2361, 264.2307, 70.0646	C_18_H_35_NO_5_	346.2588	4.62	Broussonetine C
11.130	314.2678	296.2570, 278.2464, 236.2360, 135.1156, 95.0850, 70.0646	C_18_H_35_NO_3_	314.2690	3.72	(−)-7-hydroxycassine^*∗*^
11.584	316.2833	298.2723, 280.2623, 236.2358, 198.1840, 149.1319, 95.0851, 70.0645	C_18_H_37_NO_3_	316.2846	4.18	Leptophyllin A^*∗*^
12.609	342.2987	324.2889, 306.2780, 95.0849, 81.0693, 70.0648, 55.0538	C_20_H_39_NO_3_	342.3003	4.59	(−)-7-hydroxyspectaline^*∗*^
13.231	300.2532	282.2411, 264.2309, 182.1526, 123.1158, 95.0849, 70.0648	C_17_H_33_NO_3_	300.2533	0.40	Leptophyllin B^*∗*^
13.251	358.2942	298.2723, 280.2621, 198.1835, 95.0847, 70.0646	C_20_H_39_NO_4_	358.2952	2.75	3-*O*-acetylleptophyllin A^*∗*^
14.044	300.2890	282.2772, 264.2668, 236.2355, 182.1894, 95.0849, 70.0647	C_18_H_37_NO_2_	300.2897	2.45	(−)-6-iso-carnavaline^*∗*^
14.385	298.2727	280.2627, 252.2303, 198.1837, 149.1310, 123.1158, 95.0848, 70.0645	C_18_H_35_NO_2_	298.2741	4.55	(−)-cassine^*∗*^
16.015	328.3209	310.3082, 292.2978, 264.2665, 210.2197, 95.0848, 70.0645	C_20_H_41_NO_2_	328.3210	0.32	(−)-spectalinine^*∗*^
16.419	326.3045	308.2932, 280.2612, 226.2148, 123.1156, 95.0848, 70.0645	C_20_H_39_NO_2_	326.3054	2.62	(−)-spectaline^*∗*^
17.693	368.3139	326.3025, 308.2928, 226.2136, 95.0848, 70.0645	C_22_H_41_NO_3_	368.3159	3.04	(−)-3-*O*-acetylspectaline^*∗*^
18.166	402.2986	280.2620, 252.2302, 212.1992, 184.1668, 149.1310, 70.0645	C_25_H_39_NO_3_	402.3003	4.15	3(R)-benzoyl-2-(R)-methyl-6(R)-11′-oxododecyl)-piperidine^*∗*^
18.467	300.2893	282.2772, 264.2666, 238.2507, 198.2194	C_18_H_37_NO_2_	300.2897	1.35	Sphingosine
28.349	582.5430	564.5322, 546.8983, 298.2725, 280.2618, 70.0644	C_36_H_71_NO_4_	582.5456	4.44	N-(2-hydroxy-docosanoyl)-tetradecasphing-4-enine
28.631	562.5163	453.3398, 340.2575, 264.2669, 69.0691	C_36_H_67_NO_3_	562.5194	5.46	N-(13Z-docosenoyl)-4E,6E-tetradecasphingadienine
29.616	538.5174	520.5056, 264.2669, 114.0900, 70.0643	C_34_H_67_NO_3_	538.5194	3.66	N-palmitoylsphingosine
31.276	608.5642	548.5366, 352.3180, 292.2976, 224.2333, 70.0643	C_38_H_73_NO_4_	608.5612	−4.87	N-(2-hydroxy-tetracosanoyl)-4E,6E-tetradecasphingadienine

^*∗*^Compounds identified in both ethanolic and water extracts.

**Table 3:** LC-MS/MS analysis of the water extract of *C. spectabilis* flower.

RT^a^ (mins)	(M + H)^+^	Product ions (m/z)	Formula	Exact mass	Diff (ppm)	Proposed compounds
3.499	130.0858	103.8087, 84.0801, 67.0534, 56.0488	C_6_H_11_NO_2_	130.0863	3.50	Pipecolic acid^*∗*^
6.113	216.1593	198.1475, 180.1370, 152.1421, 123.0797, 95.0848, 81.0692, 70.0646, 55.0537	C_11_H_21_NO_3_	216.1594	0.56	5-(5-hydroxy-6-(hydroxymethyl) piperidin-2-yl)pentan-2-one
7.328	230.1747	212.1631, 194.1529, 95.0845, 70.0645, 67.0537	C_12_H_23_NO_3_	230.1751	1.61	7-(5,6-dihydroxypiperidin-2-yl)heptan-2-one^*∗*^
8.494	244.1905	226.1787, 208.1681, 185.0064, 109.1003, 81.0691, 70.0645	C_13_H_25_NO_3_	244.1907	0.90	7-(5-Hydroxy-6-(hydroxymethyl) piperidin-2-yl)heptan-2-one^*∗*^
9.107	332.2790	314.2672, 296.2570, 236.2351, 135.1152, 95.0847, 70.0646	C_18_H_37_NO_4_	332.2795	1.61	Batzellaside A^*∗*^
10.536	314.2687	296.2569, 280.2618, 236.2358, 208.2046, 161.1310, 135.1157, 95.0849, 70.0646	C_18_H_35_NO_3_	314.2690	0.86	(−)-7-hydroxycassine^*∗*^
11.487	316.2838	298.2723, 280.2620, 236.2358, 198.1836, 149.1313, 121.1002, 95.0849, 70.0646	C_18_H_37_NO_3_	316.2846	2.59	Leptophyllin A^*∗*^
12.571	340.2839	322.2723, 95.0851, 70.0645	C_20_H_37_NO_3_	340.2846	2.12	3-*O*-acetylcassine
12.728	342.2992	324.2878, 306.2774, 121.1004, 95.0849, 70.0646	C_20_H_39_NO_3_	342.3003	3.13	(−)-7-hydroxyspectaline^*∗*^
13.097	300.2525	282.2407, 264.2302, 236.2350, 182.1524, 147.1153, 123.1155, 95.0848, 70.0645, 55.0536	C_17_H_33_NO_3_	300.2533	2.73	Leptophyllin B^*∗*^
13.116	358.2942	298.2724, 280.2619, 236.2341, 198.1842, 135.1150, 95.0849, 70.0647	C_20_H_39_NO_4_	358.2952	2.75	3-*O*-acetylleptophyllin A^*∗*^
13.718	300.2890	282.2772, 264.2668, 236.2356, 182.1888, 149.1312, 121.1000, 95.0850, 70.0646	C_18_H_37_NO_2_	300.2897	2.45	(−)-6-iso-carnavaline^*∗*^
14.233	298.2730	280.2627, 252.2306, 198.1837, 149.1311, 123.1158, 95.0848, 70.0645	C_18_H_35_NO_2_	298.2741	3.54	(−)-cassine^*∗*^
15.165	326.3046	308.2923, 290.2813, 266.2462, 192.1729, 95.0846, 70.0644	C_20_H_39_NO_2_	326.3054	2.32	6-iso-spectaline
16.172	328.3199	310.3082, 292.2978, 264.2665, 236.2354, 210.2199, 149.1309, 95.0848, 70.0645	C_20_H_41_NO_2_	328.321	3.37	(−)-spectalinine^*∗*^
16.733	326.3043	308.2932, 280.2612, 226.2148, 177.1620, 149.1308, 123.1156, 95.0848, 70.0644	C_20_H_39_NO_2_	326.3054	2.32	(−)-spectaline^*∗*^
17.69	368.3148	308.2928, 280.2605, 123.1155, 95.0847, 70.0647	C_22_H_41_NO_3_	368.3159	3.04	(−)-3-*O*-acetylspectaline^*∗*^
17.886	318.2992	300.2878, 256.2617, 88.0745, 70.0643, 57.0692	C_18_H_39_NO_3_	318.3003	3.36	Phytosphingosine
18.058	404.3145	282.2771, 264.2667, 196.2041, 70.0645	C_25_H_41_NO_3_	404.3159	3.51	6-(10-hydroxy-9-methylundecyl)-2-methylpiperidin-3-yl benzoate
18.424	402.2987	280.2620, 212.1992, 70.0644	C_25_H_39_NO_3_	402.3003	3.90	3(R)-benzoyl-2-(R)-methyl-6(R)-11′-oxododecyl)-piperidine^*∗*^

^*∗*^Compounds identified in both ethanolic and water extracts.

## Data Availability

The data used to support the findings of this study are available upon reasonable request from the corresponding author.
